# Drug-Induced Erythema Multiforme

**DOI:** 10.1155/2023/8706006

**Published:** 2023-10-23

**Authors:** Peeyush Shivhare, Naqoosh Haidry, Anka Sharma, Deepali Agrawal, Abhishek Gupta, Shalini Subramanyam

**Affiliations:** ^1^Department of Dentistry, All India Institutes of Medical Sciences, Patna 801507, India; ^2^Department of Oral Medicine and Radiology, Dental Institute, Rajendra Institute of Medical Sciences, Ranchi 834009, India; ^3^Oracle CBCT, Oral Medicine and Radiology, Faridabad 121003, India; ^4^Department of Oral Medicine and Radiology, School of Dental Sciences, Chitwan Medical College, Bharatpur 44207, Chitwan, Nepal; ^5^Private Dental Clinic, Bangalore 560038, India

## Abstract

Erythema multiforme (EM) is an acute inflammatory, mucocutaneous, psychosomatic, and vesiculobullous condition that varies from minor to major forms. The acral distribution of target lesions is a characteristic of this condition. The aetiology of erythema multiforme is multifactorial. 90% of the cases are triggered by a herpes infection, whereas 10% occur secondary to drug intake. The offending drugs include nonsteroidal anti-inflammatory drugs, antibiotics, and anticonvulsants. The present case series discusses four cases of drug-induced erythema multiforme and their management.

## 1. Introduction

Erythema multiforme (EM) is an acute inflammatory, mucocutaneous, psychosomatic, and vesiculobullous condition that varies from minor to major forms [1]. The name “multiforme” denotes its multiple clinical presentations varying from macules, papules, and vesicles to ulcers. The classical feature of this disease is the acral distribution of target lesions, characterized by concentric rings surrounding a darker centre. These lesions can be typical (three concentric rings) or atypical (two concentric rings) [1, 2]. Based on mucous membrane involvement, EM can be classified as EM minor (no mucosal involvement) and EM major (with mucosal involvement). The most commonly affected mucosal sites are the lips, tongue, and oral mucosa. Ocular and genital mucous membrane involvement has also been reported [1, 3]. Previously, EM was considered a part of the spectrum that involved Stevens–Johnson syndrome and toxic epidermal necrolysis; however, studies have revealed that these disorders are significantly distinct and should not be clubbed together [4].

EM has a multifactorial aetiology; however, most EM (90%) cases report infection, especially herpes (HSV-1/2), as the triggering agent. Drug-induced EM accounts for <10% of all cases [5]. In the present case series, we describe four cases of drug-induced EM and their management.

## 2. Case 1

**Figure 1:**
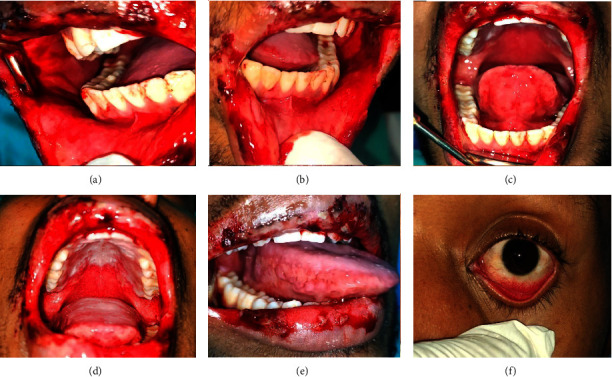
Case 1: multiple bleeding erosive areas involving buccal mucosa, labial mucosa, ventral surface of the tongue, palate, and lateral border of the tongue (a–e). (f) Erythematous areas were also seen on the lower conjunctiva and lower palpebral fissures.

A 23-year-old male presented with painful bleeding ulcers in the mouth and difficulty swallowing for two days. Three days before the presentation, he had self-medicated with paracetamol 500 mg for fever. On the second day of medication, he suddenly developed ulcers on the lips and in the mouth. His personal and family histories were noncontributory. On general physical examination, he was febrile (99.5°F) and had minor erosive areas on both thighs. Extraoral examination of the lesion revealed diffused erosive areas on the lips and corners of the mouth with bleeding and crustation. Erythematous areas were also observed in the lower conjunctiva and lower palpebral fissures. On intraoral examination, ill-defined erosive areas were noted in the oral cavity, including the tongue, upper/lower labial mucosa, bilateral buccal mucosa, hard and soft palates, and the anterior pharyngeal wall. Active bleeding was observed in these lesions (Figure 1).

**Figure 2:**
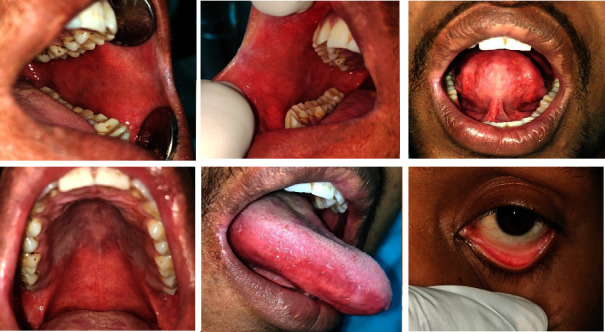
Case 1: complete healing of the lesions.

**Figure 3:**
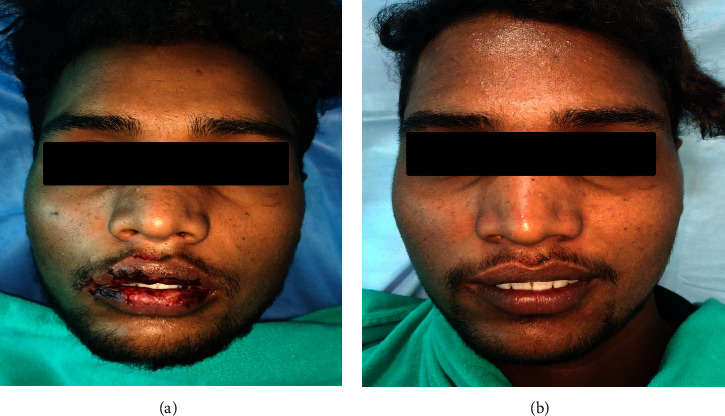
Case 1: (a) pretreatment pic and (b) post treatment pic.

Based on the patient's history and clinical examination findings, he was diagnosed with drug-induced EM. The differential diagnoses included thrombocytopenic purpura and mucous membrane pemphigoid. He received 2 ml of dexamethasone intravenously, followed by prednisolone 40 mg for ten days, tapered over two weeks. Three days later, he reported moderate ulcer relief. However, he developed fluid-filled vesicles in the right corner of his mouth the following day. Serology tests confirmed that he was positive for HSV, and there was an increase in the antibody titer; thus, acyclovir 400 mg, five times a day, was added to the regimen. A one-month follow-up revealed complete remission (Figures 2 and 3).

## 3. Case 2

**Figure 4:**
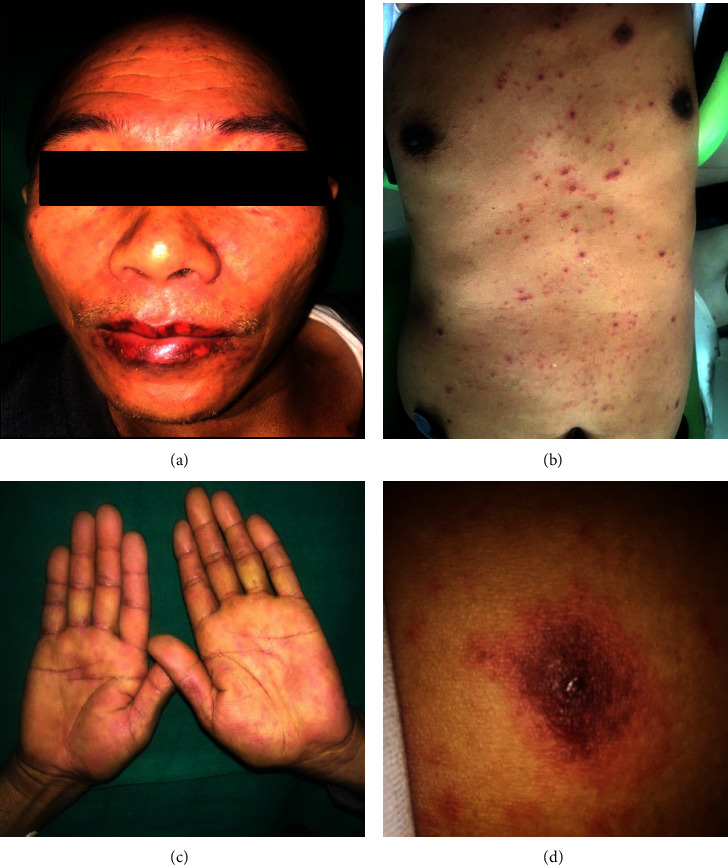
Case 2: extraoral lesions associated with intraoral lesions. (a, c) Multiple erythematous rashes all over the face and palms. (b, d) Multiple “targetoid lesions” were noted on the chest, waist, and back region.

**Figure 5:**
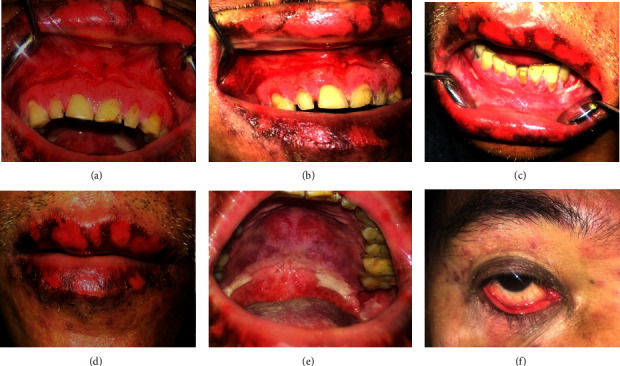
Case 2: multiple bleeding erosive areas involving buccal mucosa, labial mucosa, ventral surface of the tongue, palate, and lateral border of the tongue (a–e). (f) Erythematous areas were also seen on the lower conjunctiva and lower palpebral fissures.

**Figure 6:**
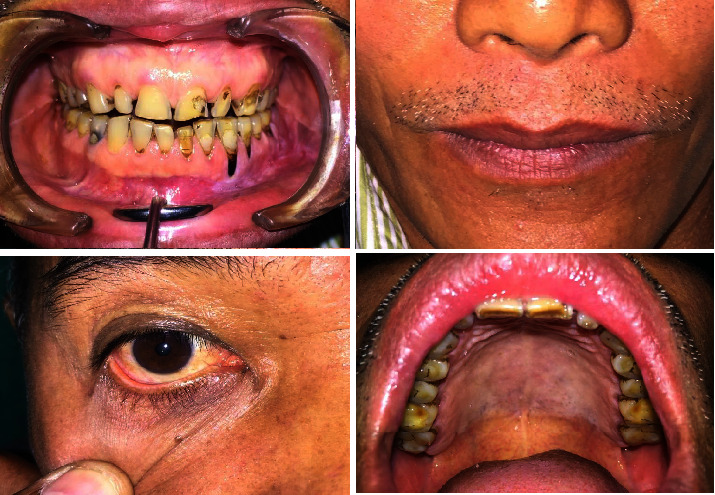
Case 2: complete healing of the lesions.

**Figure 7:**
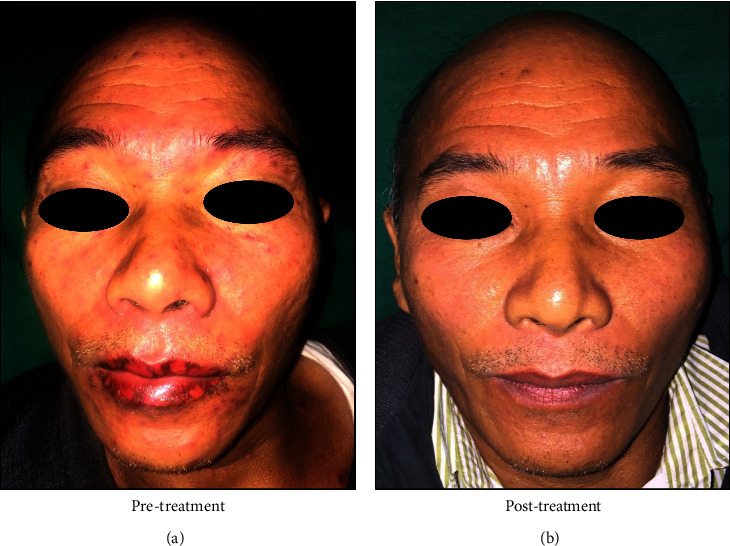
Case 2: (a) pretreatment pic and (b) post treatment pic.

A 49-year-old male presented with ulceration of the gums and rashes all over the body for two days. Two days before the presentation, he was diagnosed with trigeminal neuralgia and was prescribed carbamazepine 100 mg three times a day. Soon after taking the medication, he developed rashes, which worsened after the next dose. He had no clinical history of such lesions. His personal and family histories were noncontributory. Extraoral examination revealed multiple erythematous rashes on the face and palms. Multiple targetoid lesions were noted on the chest, waist, and the back region (Figure 4). Erythematous areas were observed in the lower conjunctiva and lower palpebral fissures. Intraoral examination revealed multiple erosive areas on the bilateral vestibules, gingiva of both arch, and the palate (Figure 5). Nikolsky's sign was negative. On palpation, the ulcers were highly tender with profuse bleeding. Serological tests were negative for HSV. He was diagnosed with drug-induced EM and managed with prednisolone 40 mg once daily for 10 days, which was subsequently tapered (30 mg, 20 mg, 10 mg, 5 mg, and 2.5 mg) over four weeks. Fluconazole tablets (150 mg for 3 days) were also advised to avoid superadded candidal infection. A one-month follow-up revealed complete remission (Figures 6 and 7).

## 4. Case 3

**Figure 8:**
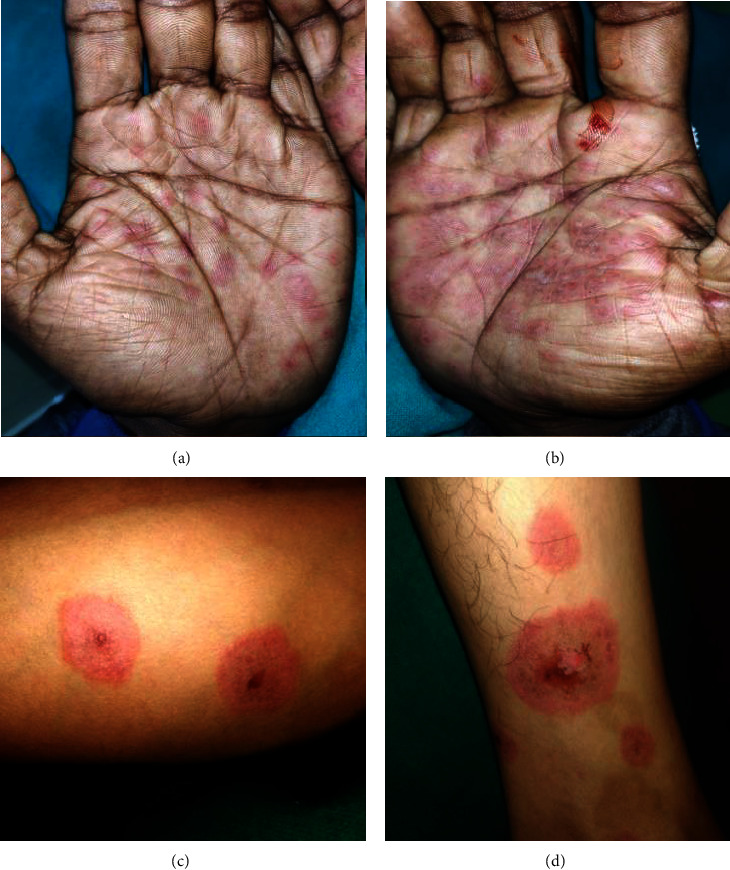
Case 3: Typical “Target lesions” (a, b) on palms (c) on forearm and (d) on leg.

**Figure 9:**
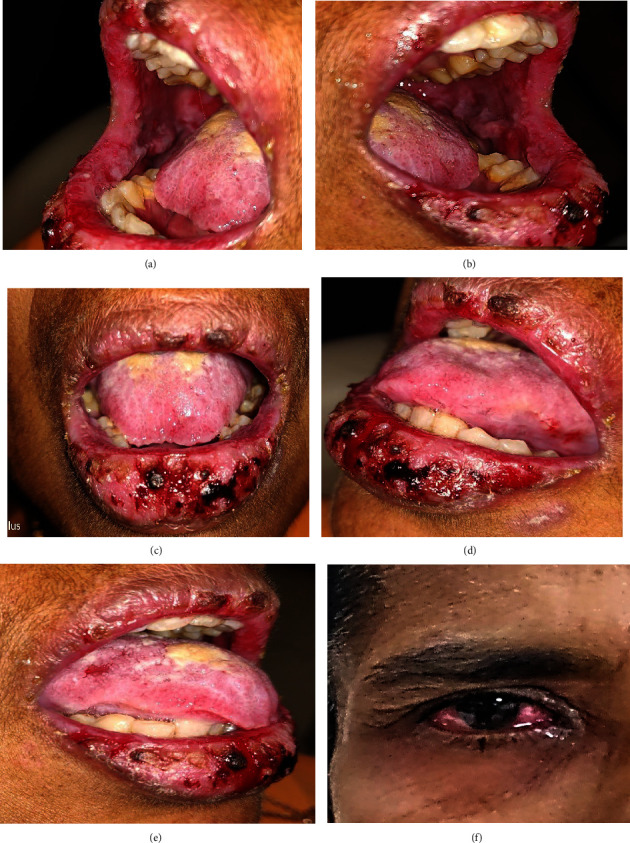
Case 3: multiple bleeding erosive areas involving buccal mucosa, labial mucosa, ventral surface of the tongue, palate, and lateral border of the tongue. (a–e). (f) Erythematous areas were also seen on the lower conjunctiva and lower palpebral fissures.

**Figure 10:**
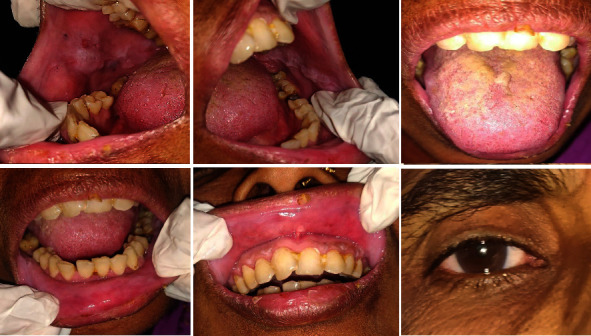
Case 3: complete healing of the lesions.

**Figure 11:**
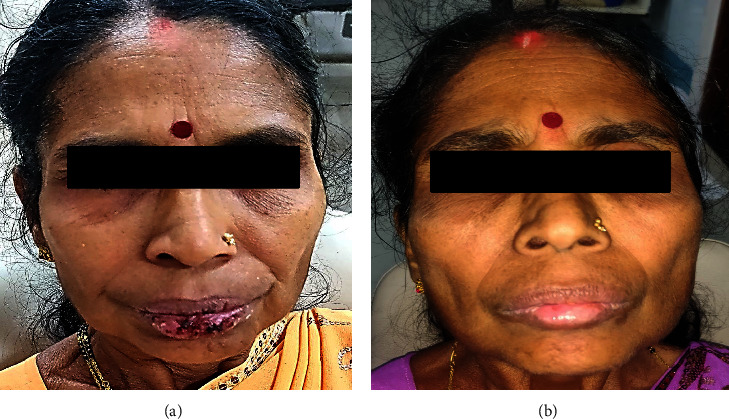
Case 3: (a) pretreatment pic and (b) post treatment pic.

A 63-year-old female presented with ulcerations in the mouth, pain, and inability to eat for one week. She had a past history of diarrhoea and abdominal pain for two weeks, for which she was administered cefixime 200 mg and diclofenac sodium 50 mg. Within 12 hours, she developed multiple ulcerations of the lips and mouth. She had visited a local dentist, where she was prescribed a local anaesthetic gel and amoxicillin 500 mg, which did not provide any relief. Typical target lesions were evident on the palms, legs, and feet (Figure 8). Multiple ulcerations with crustation and bleeding points were evident on both the upper and lower lips. Erythematous areas were also observed in the conjunctiva and lower palpebral fissures. The intraoral examination revealed extensive irregular ulcerations with sloughing of the entire oral mucosa (Figure 9). Nikolsky's sign was negative. Serological tests were negative for HSV. She was also administeredprednisolone 40 mg once daily for 10 days and tapered over the next 28 days (30 mg, 20 mg, 10 mg, 5 mg, and 2.5 mg). A one-month follow-up revealed complete remission (Figures 10 and 11).

## 5. Case 4

**Figure 12:**
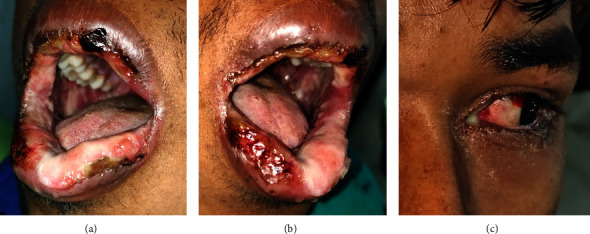
Case 4: (a, b) multiple erosive areas involving buccal mucosa, labial mucosa, and palate. (c) Erythematous areas were also seen on the lower conjunctiva and lower palpebral fissures.

A 27-year-old male presented with a complaint of painful ulcerations in the mouth for one week. He had a history of headaches, for which he took paracetamol 500 mg from a local drug dealer. Within 24 hours, he developed multiple ulcerations on the lips and mouth. Multiple ulcerations with crustations were evident on the upper and lower lips. Erythematous areas were observed in the conjunctiva and lower palpebral fissures. The intraoral examination revealed extensive irregular ulcerations with sloughing of the entire oral mucosa (Figure 12). Nikolsky's sign and serological tests for HSV were negative. He was managed with methylprednisolone 16 mg once daily for 10 days, which was tapered over the next 21 days (8 mg, 4 mg, and 2 mg). He was also advised to apply a topical 0.1% triamcinolone acetonide ointment, and fluconazole tablets (150 mg tablet for three days) were administered to avoid candida superinfection. He informed about complete remission in three weeks by telephone communication (follow-up images could not be taken due to the COVID-19 pandemic).

## 6. Discussion

EM was first reported in 1,846 by Bateman and Bulky, followed by Hebra in 1866 [6]. Pathogenetically, the aetiologies of Herpes-associated EM and drug-induced EM are different. Herpes-associated EM (HAEM) is a hypersensitivity reaction in which CD4+Th1 cells produce IFN-*γ* (interferon-gamma). IFN-*γ* initiates an inflammatory cascade that includes expression of IFN- *γ* induced genes, increased sequestration of circulating leukocytes, monocytes and natural killer (NK) cells, and the recruitment of autoreactive T-cells. The cytotoxic T-cells, NK cells, and chemokines ultimately result in keratinocytes damage [7]. In contrast, in drug-induced EM (DIEM), the destruction is mediated by the tumor necrosis factor alpha (TNF-*α*) [7]. In addition, in patients with DIEM, there is abnormal metabolism of the offending drug, which increases the production of toxic metabolites via the cytochrome-P450 pathway [8]. The most common offending drugs reported are nonsteroidal anti-inflammatory drugs (NSAIDs), antibiotics, and anticonvulsants [9]. In this study, we are reporting paracetamol, carbamazepine, cefixime, and diclofenac as the offending drugs.

Clinically, HAEM and DIEM are indistinguishable. Both entities present extensive, ill-defined, erosive areas involving the skin and mucous membranes, along with target lesions. However, in HAEM, the viral culture tests positive (ELISA or PCR for HSV-1/2). The pathognomonic feature of DIEM is the temporal relationship between drug intake and the appearance of lesions. Clinicians should differentiate DIEM from other adverse reactions, especially fixed-drug reaction (FDR). FDR tends to occur at the same site even after repeated insults, whereas DIEM can spread to involve multiple sites. In addition, FDR does not include target lesions.

Soares and Sokumbi suggested that the offending drug must be discontinued, as a primary treatment of DIEM [2]. In patients with mild mucosal involvement and mild discomfort, topical steroids along with antiseptics and antihistamines are recommended. In patients with moderate mucosal involvement, systemic steroids should be initiated at a starting dose of 40–60 mg tapered over 2 weeks [4, 10]. Antiseptics and antihistamines should also be included. Patients with ocular involvement require an ophthalmic consultation (antibiotic/steroid eye drops) to avoid future complications. In severe cases, hospitalization is recommended for electrolyte repletion and intravenous steroid administration [4]. Cases that are nonresponsive to steroids can be managed successfully with azathioprine, apremilast, cyclosporine, mycophenolate mofetil, thalidomide, adalimumab, hydroxychloroquine, levamisole, and dapsone [10, 11].

In this report, all patients had moderate mucosal and ocular involvement. Thus, systemic steroids (prednisolone 40 mg), antihistamines (benzydamine hydrochloride 0.15%), and an antiseptic mouthwash (betadine) were administered. An ophthalmic consultation was also conducted when betamethasone eyedrops were prescribed. In case 1, acyclovir was initiated because of superimposed herpes infection. All three patients responded well to systemic steroids, which were later tapered over 2 weeks.

Because of the temporal relationship between drug intake and the development of the lesions, none of our case was investigated for the presence of Mycoplasma. Historically, dermatologic/mucosal manifestation of mycoplasma and other infectious organisms was considered as the spectrum of erythema multiforme (EM), Stevens–Johnson syndrome (SJS), and toxic epidermal necrolysis (TEN). However, recent literatures suggested it as separate clinical entity and classified under broad category of reactive infectious mucocutaneous eruption (RIME) [12].

Reactive infectious mucocutaneous eruption describes an eruption of prominent mucositis with/without cutaneous involvement triggered by *Mycoplasma pneumoniae* (MP) and other infectious organism [13]. *Mycoplasma pneumoniae* infection is associated with mucocutaneous eruptions typically with prominent mucosal involvement and is termed as *Mycoplasma pneumoniae*-induced rash and mucositis (MRIM) as variant of RIME. The criteria for RIME include the following [14]: (a) evidence of an infectious trigger based on symptoms, imaging studies, or lab tests and (b) at least two of (i) vesiculobullous/atypical target skin lesions affecting <10% OR (ii) erosive mucositis involving two or more mucous membranes OR (iii) noncontributory medication history.

### 6.1. Limitations

All of our cases were diagnosed solely based on the temporal relationship between drug intake and the development of the lesions. The cases were not tested for the presence of mycoplasma/other associated viruses and in vivo or in vitro drug testing to confirm drug causality. We also did not perform a biopsy or immunofluorescence test to rule out other causes of mucositis such as mucous membrane pemphigoid. Thus, the authors suggest strongly performing these investigations to give the diagnosis of drug-induced erythema multiforme.

## 7. Conclusion

DIEM is a less commonly reported variant of EM, and the offending drugs are mostly NSAIDs, antibiotics, and anticonvulsants. The oral mucosa is most commonly involved, especially the lips, tongue, and buccal mucosa. A temporal relationship exists between recent drug intake and the appearance of symptoms in EM. For the management of EM, the offending drug should be immediately discontinued and steroids should be initiated depending on the extent and severity of the involvement.

## Data Availability

The data used to support the findings of the study are included within the article and are also available via email from the corresponding author.

## Abbreviations

EM: Erythema multiforme
HSV: Herpes simplex virus
HAEM: Herpes-associated EM
IFN-*γ*: Interferon-gamma
DIEM: Drug-induced EM
NSAIDs: Nonsteroidal anti-inflammatory drugs
ELISA: The enzyme-linked immunosorbent assay
PCR: Polymerase chain reaction
FDR: Fixed-drug reaction
SJS: Stevens–Johnson -syndrome
TEN: Toxic epidermal necrolysis
RIME: Reactive infectious mucocutaneous eruption
MRIM: *Mycoplasma pneumoniae-*induced rash and mucositis.
